# Literature review and case report of septic arthritis and purpura fulminans leading to a child limb amputation as chickenpox complications

**DOI:** 10.1097/MS9.0000000000002412

**Published:** 2024-08-02

**Authors:** Bassel Hijazi, Effat Nairoukh, Razan M. Yahya, Fawzy M. Abunejma

**Affiliations:** Al Quds University School of Medicine, Faculty of Medicine, Al Quds University, Palestine

**Keywords:** chickenpox, pediatrics, purpura fulminans, septic arthritis, varicella

## Abstract

**Introduction and significance:**

Chickenpox, induced by the varicella-zoster virus (VZV), generally presents with an itchy rash and fluid-filled blisters. While complications such as pneumonia and sepsis are well-documented, occurrences of septic arthritis and purpura fulminans are exceedingly rare. Septic arthritis following varicella infection is infrequently reported and often attributed to *Staphylococcus aureus*. Purpura fulminans encompasses disorders characterized by rapidly progressing purpuric lesions, often fatal and associated with consumptive coagulopathy.

**Case presentation:**

The authors present the case of an 8-year-old boy diagnosed with chickenpox who concurrently developed severe left knee pain, erythema, and swelling indicative of septic arthritis, along with a single pustular lesion on his right foot that progressed to purpura fulminans. Laboratory investigations revealed elevated inflammatory markers. Knee ultrasound findings were consistent with septic arthritis, corroborated by synovial fluid analysis. Immediate initiation of empiric antibiotics was undertaken. Further investigation disclosed unusual coagulation parameters, positive autoantibodies, and reduced protein S levels. Treatment included anticoagulation, immunomodulation, and ultimately, amputation.

**Clinical discussion:**

This rare case underscores the complexity of varicella-related complications, representing the first documented instance of simultaneous septic arthritis and purpura fulminans in a pediatric patient. It highlights the necessity of a multidisciplinary approach for accurate diagnosis and management, emphasizing the importance of recognizing rare complications to improve patient outcomes.

**Conclusion:**

This case exemplifies the complexity of varicella-associated complications, showcasing a rare simultaneous occurrence of septic arthritis and purpura fulminans in a pediatric patient. It underscores the importance of a thorough understanding and collaborative management approaches for timely intervention and enhanced clinical outcomes.

## Introduction

Highlights

- Novel results: First documented instance of simultaneous septic arthritis and purpura fulminans in pediatric varicella infection.
- New methods: Utilization of multidisciplinary approach for precise diagnosis and management.
- Key findings: Unusual coagulation parameters and positive autoantibodies observed in the patient.
- Breakthrough insight: Highlighting the complexity and rare complications of varicella infection.
- Implications: Emphasizing the need for timely intervention and collaborative management for improved outcomes.

Chickenpox is an infection caused by the varicella-zoster virus (VZV). It causes an itchy rash with small, fluid-filled blisters. Chickenpox is highly contagious to people who haven’t had the disease or been vaccinated against it. It could be complicated by pneumonia, sepsis, encephalitis, and with the rarest complication septic arthritis and purpura fulminans^1^. Septic arthritis that develops following varicella is rarely seen. Twenty-nine patients reported in the literature with a 30% frequency of bacterial growth in the synovial fluid cultures of patients (mainly negative growth). The most frequent agent in septic arthritis is *Staphylococcus aureus*, followed by Group A streptococci, *Streptococcus pneumoniae*, and *Kingella kingae*. Although primary viral agent-driven septic arthritis is very rare, they can be seen in the form of an immune-mediated reaction, especially following the live vaccines. Purpura fulminans is a descriptive term depicting a heterogeneous group of disorders characterized by rapidly progressive purpuric lesions, which may develop into extensive areas of skin necrosis and peripheral gangrene. The disorder is associated with laboratory evidence of consumptive coagulopathy. The histopathologic features are widespread thrombosis of the dermal capillaries and venules with hemorrhagic infarction of the surrounding tissues. The condition is often fatal, and survivors may have considerable morbidity related to the loss of digits, limbs, or areas of skin^2–4^.

### Case report

We report the case of an 8-year-old boy admitted to our hospital due to severe progressive left knee pain for 2 days, following three days of multiple crusted vesicular lesions distributed across his body, diagnosed as chickenpox. The pain was associated with erythema, warmth, swelling, and resistance to knee flexion or extension. Physical examination revealed an irritable patient with notable crusted vesicular lesions and a high fever of 38.5°C as documented by the doctor. Laboratory tests demonstrated elevated WBC of 14 000, neutrophils at 71.4%, lymphocytes at 14%, hemoglobin at 10 g/dl, CRP at 212 mg/l, ESR at 83 mm/h two hours after admission, uric acid at 2.6 mg/dl, with platelet and coagulation profiles within normal ranges, and a negative Brucella test. Ultrasound of the knee revealed a large effusion of 40 ml with internal echoes extending into the suprapatellar bursa with medial suprapatellar plica, consistent with septic arthritis. Aspiration was performed, followed by empiric antibiotics (ceftriaxone 75 mg/kg/day IV for 4 days and vancomycin 20 mg/kg/day IV for 7 days). Chemical and cytological analysis of synovial fluid showed a turbid yellow color, no crystals, pH 7, WBC 14×10^9^/l, neutrophils at 80%, lymphocytes at 20%, and culture returned negative (no growth).

**Figure 1:**
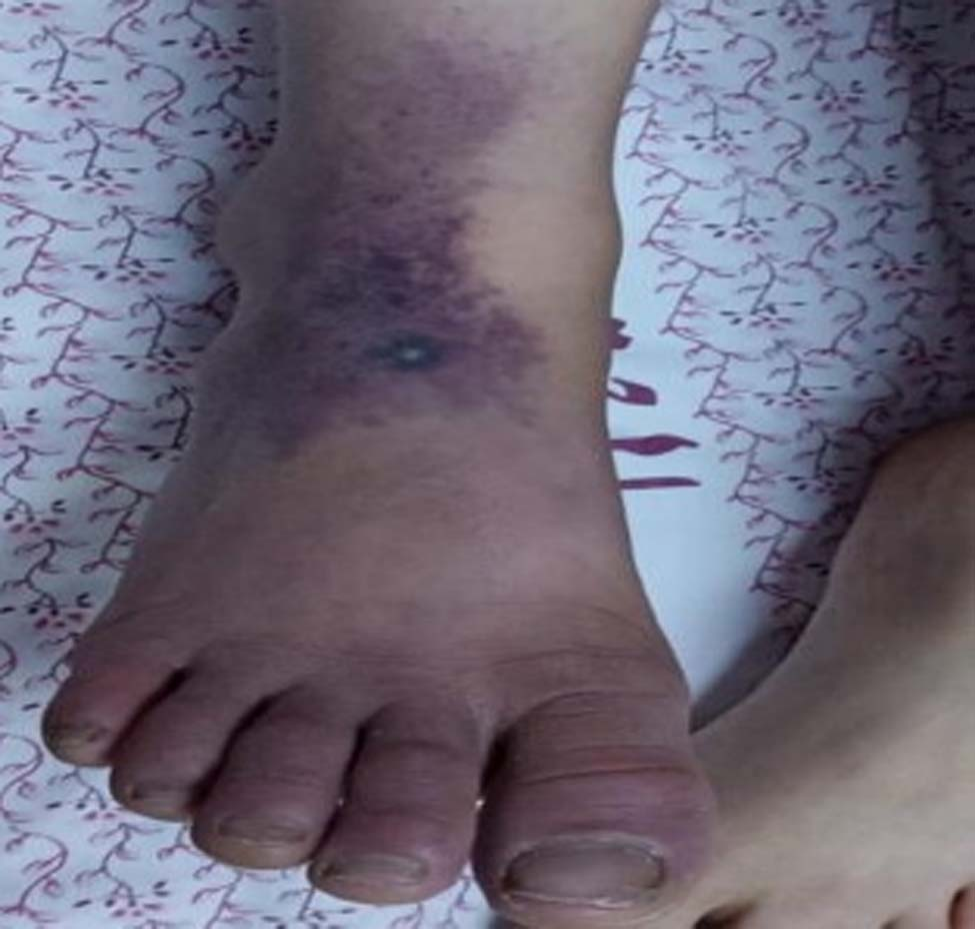
Single pustular lesion with a dark base on the right foot.

**Figure 2:**
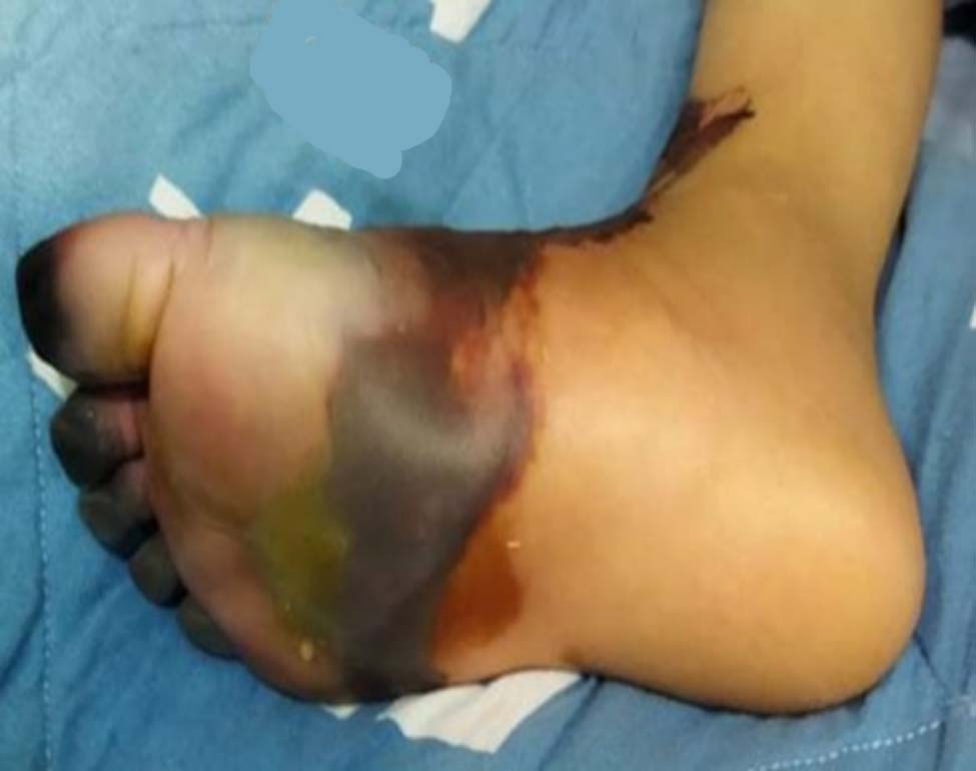
Bluish discoloration spreading from the lesion, involving the ankle and proximal tibia, with severe pain.

**Figure 3:**
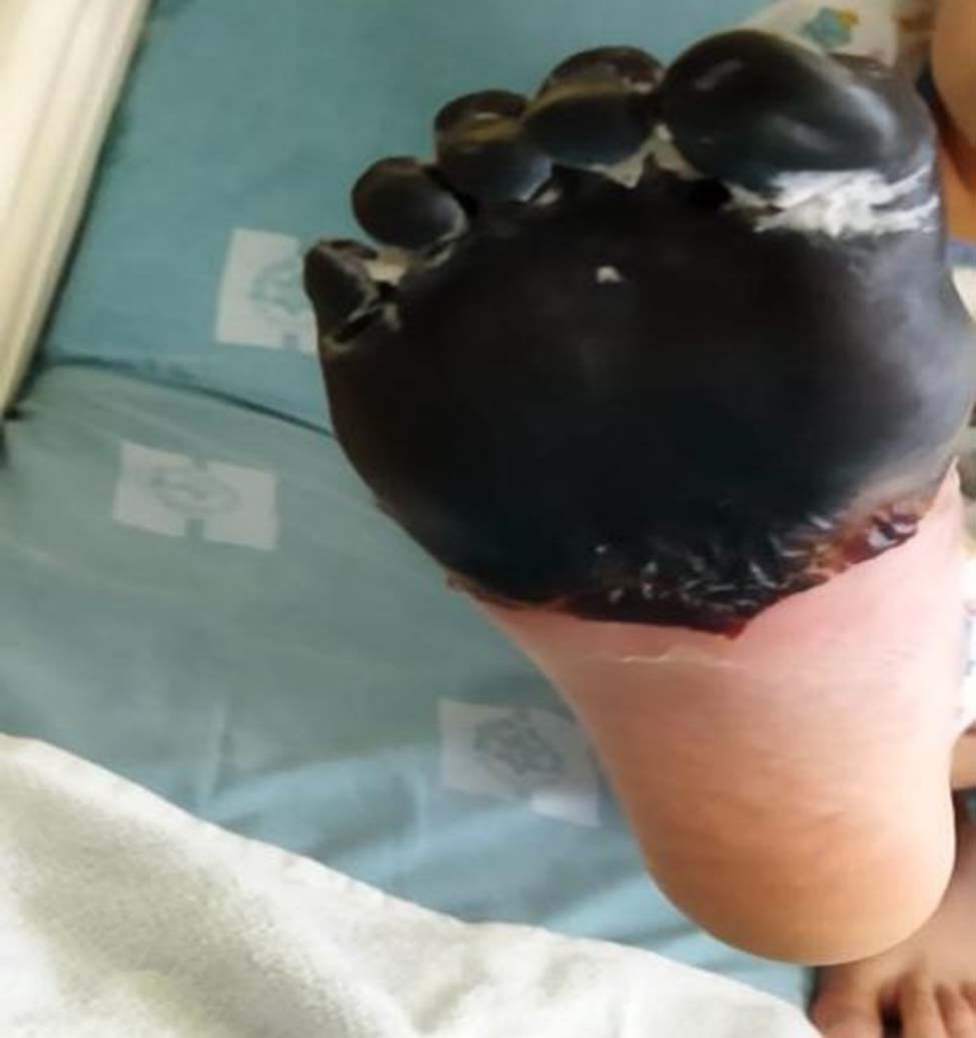
Increased edema and tense tenderness in the foot, with darker lesions as symptoms progressed.

**Figure 4:**
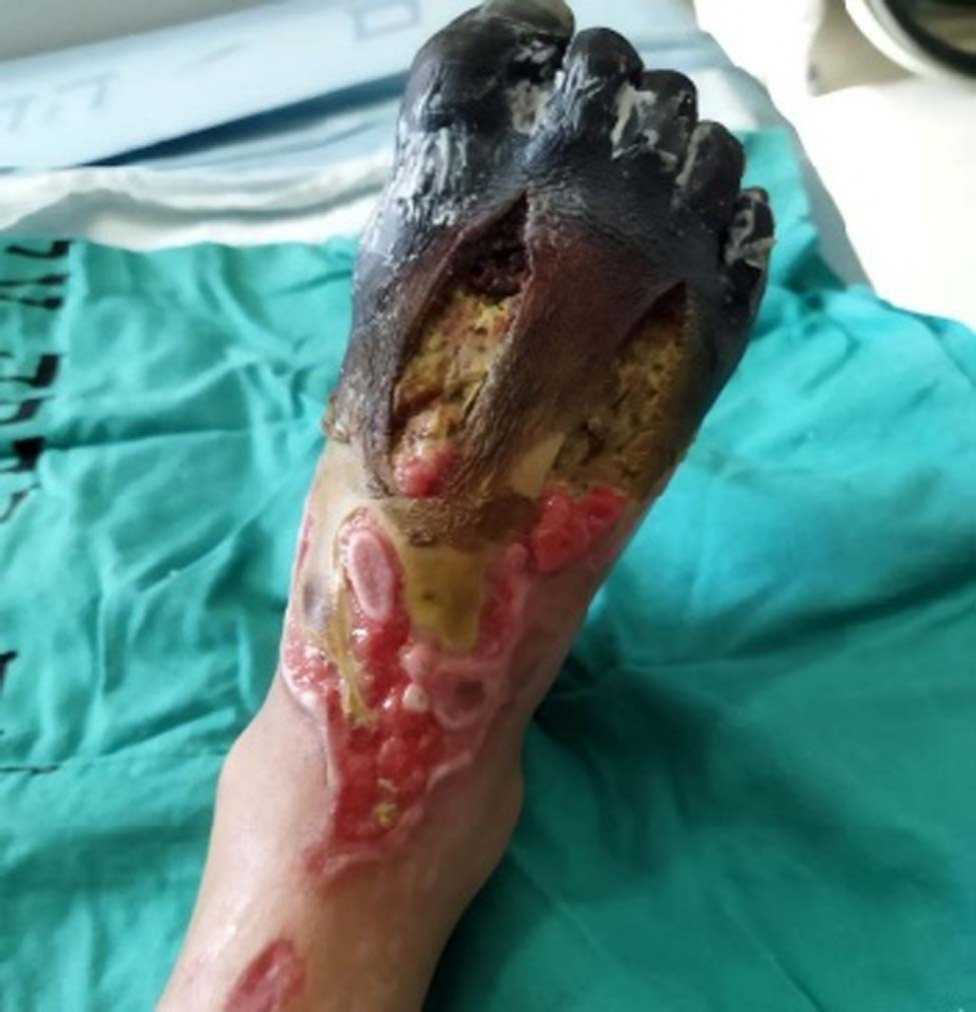
Increased edema and tense tenderness in the foot, with darker lesions as symptoms progressed.

During his hospital stay for septic arthritis treatment, on the sixth day, he suddenly developed a single pustular lesion on a dark base on the dorsum of his right foot (Fig. 1). The lesion was surrounded by an area of bluish discoloration that progressively enlarged, involving the ankle and the proximal third of the tibia, accompanied by extreme pain (Fig. 2). His vital signs were stable with a temperature of 37.5°C. Upon examination, the right foot became cold and edematous over time, with palpable peripheral pulses. Coagulation profile showed D-dimer at 70 000 ng/ml FEU (normal range for his age 100–560 ng/dl FEU), fibrinogen at 114 mg/dl (normal range 200–400 mg/dl), platelets at 225 mcl, and PT/PTT/INR within normal ranges. Inflammatory markers showed ESR at 65 mm/h, CRP at 162 mg/l, and WBC at 16.4×10^9^. Doppler ultrasound and multiphase CT angiography of the lower limb revealed no abnormalities with patent blood flow. The patient was started on heparin infusion (25 IU/kg/hr) and gentamicin (2 mg/kg/dose every eight hours) with no improvement. Contrarily, symptoms worsened, and the foot became more edematous with tense tenderness and darker lesions (Figs. 3,4). The following day, the patient was transferred to a more advanced tertiary center where further investigations revealed: Protein S at 12.7% (normal range 70–140%), Anti-Xa levels at 0.2 (0.6–1.0 units/ml). Antinuclear antibody (ANA), anti-DNA antibody, and antiphospholipid antibody were positive, but there was no evidence of rheumatological disease based on his normal history and current symptoms. Notably, both parents had normal protein S levels.

The child was treated with daily fresh frozen plasma, Enoxaparin IM 40 mg twice daily titrated against Anti-Xa levels, 3 units of vitamin K, and 2 courses of synthetic PG analog (Iloprost), each for 5 days. The patient experienced severe pain with a VAS score of 10/10, managed by patient-controlled analgesia (PCA) with morphine throughout his hospital stay. Over time, calcium and phosphate levels (previously normal) increased: Ca at 3.82 mmol/l (normal range 2.2–2.7 mmol/l), phosphate at 2.1 mmol/l (normal range 1.12–1.45 mmol/l), with normal kidney function (Cr at 31 umol/l, normal range 25–42 umol/l), indicating necrosis. Consequently, surgical debridement to clear necrotic tissue was performed. Despite this, the purpuric lesion progressed, leading to extensive toe necrosis with dry gangrene, necessitating a below-knee amputation based on orthopedic consultation. The surgery was completed without complications. Protein S levels improved to 56% U/dl and Anti-Xa to 0.7 units/ml after 5 weeks of treatment. Fortunately, the child was fitted with an artificial limb and was able to walk.

### Review of literature

We initiated our work by conducting a bibliographic search in the Medline database (1946 to 1 July 2021) using the following keywords: VZV, chickenpox, varicella, varicella zoster virus, arthritis, “idiopathic purpura fulminans,” “acquired protein S deficiency,” and “anti-protein S antibodies.” The search was limited to publications in English, and the references in the articles were reviewed for all cases of varicella arthritis and purpura fulminans. Missing information was obtained from the authors. All articles that met the inclusion criteria and presented sufficient data were included. The same data were collected as would be in a retrospective case series. The demographic, clinical, and biological characteristics of patients with varicella arthritis are presented in Table 1 ^6^.

**Table 1:** Clinical and biological characteristics of patients with varicella arthritis by year of report^14^.

						Blood		Synovial fluid lymphs	
Reference	Age (year)	Sex	Joint affected	Onset^a^(day)	Duration of arthritis	WBC/mm^3^	ESR mm/1st h	WBC	Lymphs (%)
Our case	8	M	Left knee	2	< 1 month	14 000	83	14 000	20
Sibylle Bevilacqua^6^	4	F	Right hip	-6	<1 month	11 000	30	4200	49
Ward and Bishop^7^	5	F	Left knee	2	<1 month	5400	7	3850	93
Friedman and Naveh^8^	4	F	Multijoint	5	<1 month	10 500	25	ND	ND
Mulhern *et al.* ^9^	10	M	Right knee	2	<1 month	6400	ND	3600	91
Priest *et al.* ^10^	8	F	Left knee	3	<1 month	4200	27	25 000	100
Brook^11^	6	F	Bilateral knee	7	<1 month	16 700	6	51 700	15
Brook^11^	7	F	Right knee	7	<1 month	7200	25	ND	ND
Diliberti *et al.* ^12^	5	F	Right ankle	3	<1 month	13 300	35	ND	ND
Borgenicht^5^	5	M	Left knee	4	<1 month	3500	14	20 400	80
Pascual—Gomez^13^	11	F	Left knee	1	<1 month	ND	ND	6000	100
Shuper *et al.* ^14^	2.5	F	Left foot	-1	<1 month	7400	28	ND	ND
Atkinson *et al.* ^15^	6	M	Right knee	5	<1 month	5400	13	18 300	70
Younes and Freeman^16^	9	M	Left knee	4	<1 month	4700	6	7380	39
Gibson and Ogden^17^	7	F	Left knee	2	<1 month	5800	ND	3500	92
Stabile *et al.* ^18^	7	M	Bilateral knee	2	<1 month	4400	32	6700	95
Fierman^19^	1.5	M	Left ankle	-1	1 month	ND	ND	ND	ND
Fink et a1^20^	9	M	Left knee	1	in 2 month	ND	ND	ND	ND
Quintero Del Rio and Fink^21^	6	F	Multijoint	2	Arthritis for 3 months	Normal	44	7250	39
	2.5	F	Left ankle + foot	5	Arthritis for 3 months	Normal	39	4450	2
	4.5	M	Bilateral knee	2	Intermittent arthritis	ND	ND	ND	ND
	4.5	F	Left foot	7	for 3 years <I month	ND	ND	ND	ND
	5	M	Right shoulder	5	< 1 month	ND	ND	30 000	15
	1.5	M	Left ankle	0	< 1 month	11 600	3	73 500	82
	2	F	Right knee	10	Active arthritis for 6 months	ND	ND	ND	ND
	6	M	Left knee	5	Active arthritis	ND	ND	ND	ND
Stebbings *et al.* ^22^	1O	F	Right knee	5	<2 months	5900	8	1650	95
Chen *et al.* ^23^	2	F	Left knee	4	< 1 month	ND	ND	ND	ND
J B T Lim and J S Huntley^24^	1.5	M	Hip	10	< 1 month	ND	ND	ND	ND
	2.5	F	Right Hip+Knee	5	< 1 month	ND	ND	ND	ND

ERS, erythrocyte sedimentation rate; F, female; lymphs, lymphocytes; M, male; ND, not determined; WBC, white blood cell.

^a^ (-) means days before the onset of Varicella infection clinical symptoms.

The median age was 5.5 years (interquartile range: 1–11), with a predominance of girls (71.4%). All patients were immunocompetent. Joint involvement was confined to a single joint in 27 cases (93%), while 2 cases presented with involvement of more than one joint. The knee was the most commonly affected joint (18/29 cases, 62%), followed by the ankle (4/29 cases, 13.7%), the shoulder (1/29 cases, 3.4%), and the foot (3/29 cases, 10.3%). The median time to onset of arthritis was 2 days (interquartile range: 6 days before the onset of varicella infection up to 10 days after). VZV DNA was isolated by polymerase chain reaction (PCR) from synovial fluid in 3 cases. No positive viral cultures were reported. The median ESR rate was 43 mm/1st h (interquartile range: 3–83), and the median leukocyte count in synovial fluid was 7250/l (interquartile range: 1650–73 500) with a lymphocyte count of 81% (interquartile range: 2–100). Clinically, all symptoms and signs of arthritis resolved within one month in 79.3% of cases (23/29), within two months in 6.8% (2/29), and within six months in 3.4% (1/29). Finally, one child presented with chronic arthritis, and one child with intermittent arthritis in the context of a moderate varicella rash.

Regarding the literature on purpura fulminans^25^: The median age at diagnosis was 4.9 years (range, 1.5–11 years) with a male-to-female ratio of 0.52. The time to diagnosis was 7 days (range, 2–17 days). The clinical presentation was an ecchymotic or necrotic purpura of a lower limb (*n*=49; 94%), the calves only (*n*=13; 25%), an upper limb (*n*=7; 13%), the torso (*n*=11; 21%), or the genitalia (*n*=4; 8%). A viral infection was confirmed in 45 patients (86%): chickenpox (*n*=41; 78%) or Human herpesvirus 6 (HHV-6) (*n*=4; 8%). Protein S (PS) deficiency was confirmed in all patients. The median PS activity level was 4% (range, 1–28%). The median free PS antigen level was 1% (range, 1–16%), and the median total PS antigen level was 5% (range, 1–62%). Anti-PS antibodies were confirmed in 30 patients (58%). At admission, the median Antithrombin (AT) activity level was 85% (range, 45–130%), the median protein C (PC) activity level was 49.5% (range, 14–131%), the median fibrinogen level was 0.88 g/l (range, 0.1–3.4 g/l), and the median platelet count was 150×10^9^/l (range, 10–302 g/l). D-dimer levels were tested in 36 (69%) of 52 cases, and they increased in 30 (83%) of these cases.

### Treatment and outcomes

Treatments for idiopathic purpura fulminans (IPF) included intravenous (IV) heparin (*n*=51; 98%), IV fresh frozen plasma (FFP) (*n*=41; 79%), IV polyvalent immunoglobulins (Ig’s) (*n*=20; 38%), IV corticosteroids (*n*=16; 31%), plasmapheresis (*n*=13; 25%), and IV coagulation inhibitor concentrates (AT or activated PC) (*n*=12; 23%). PS levels normalized in all patients, albeit with variable time frames. PS levels began to increase at 10 days (range, 2–49 days), with a median normalization delay of 60 days (range, 6–120 days). There were no deaths, although 25 patients (47%) experienced serious thrombotic complications, including distal amputation (*n*=14; 27%) or skin necrosis with grafting (*n*=15; 29%) (four patients required both); 17 patients (33%) developed venous thromboembolism, and two patients had hemorrhages (one subarachnoid and one pulmonary alveolar).

We analyzed the relationship between several factors and the occurrence of severe complications in the overall cohort. At the time of diagnosis, the median AT activity level (79% vs. 101%; *P*<.001) and the median platelet count (51.5×10^9^/l vs. 188.5×10^9^/l; *P*<.001) were significantly lower in patients with severe complications. Univariable analyses confirmed these results with odds ratios (ORs) of 1.08 (95% CI, 1.03–1.14; *P*=.003) for the median AT activity level and 1.02 (95% CI, 1.01–1.02; *P*=.001) for the median platelet count. In multivariable analysis, a correlation was found between severe complications and the median AT activity level, with an OR of 1.07 (95% CI, 1.01–1.14; *P*=.03), and the median platelet count with an OR of 1.01 (95% CI, 1.00–1.02; *P*=.04). All results are presented in Figure 2 and Table 2.

**Table 2:** Patient characteristics, clinical presentation, type of virus, biology at diagnosis, treatment and outcomes of recent case series in comparison to literature.

	Literature	
	Case No., *n* (%)	Median (range)
Patient characteristics		
Age, year		4.9 (1.5–11)
Male sex	27 (52)	
Clinical presentation		
Time to diagnosis, day		7 (2–17)
Lower limb	49 (94)	
Calves only	13 (25)	
Extended leg	36 (69)	
Upper limb	7 (13)	
Genitalia	4 (8)	
Torso	11 (21)	
Virus		
HHV-6	4 (8)	
VZV	41 (78)	
Unknown	7 (14)	
Biology at diagnosis		
PS activity, %		4 (1–28)
PS free antigen, %		1 (1–16)
PS total antigen, %		5 (1–62)
Anti-PS antibody	30 (58)	
PC activity, %		49.5 (14–131)
Antithrombin level, %		85 (45–130)
Fibrinogen level, g/l		0.88 (0.1–3.4)
Platelet count, X10%/l		150 (10–302)
Increase in D-dimer	30/36 (83)	
Treatment		
Heparin	51 (98)	

PC, protein C; PS, protein S.

## Discussion

### Varicella complications: septic arthritis and purpura fulminans

Varicella, commonly seen in childhood, is generally a benign disease. However, its most frequent complications include skin and soft tissue infections, neurological complications, and pneumonia. Septic arthritis following varicella is rare, as is purpura fulminans; this is the first documented case in which both conditions occur in the same patient according to the literature.

### Septic arthritis

**Figure 5:**
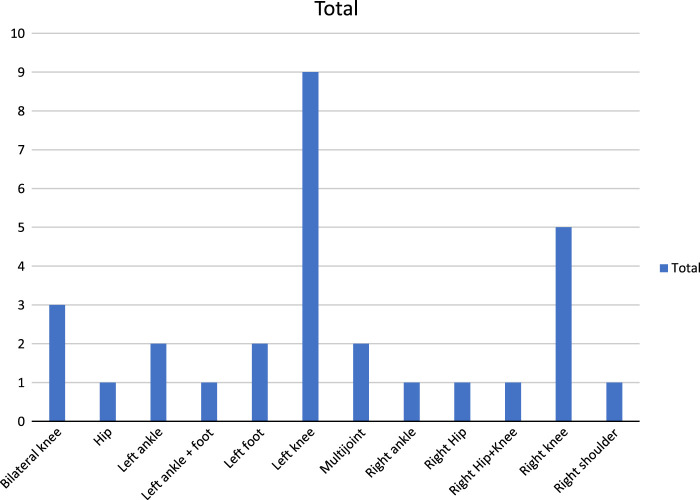
Literature-reported locations of septic arthritis post-varicella infection (Y=number of reported events).

There have been 29 reported cases of joint involvement due to varicella (Fig. 5). This involvement can occur due to direct viral invasion, resulting in aseptic arthritis, or as a secondary bacterial infection. Differentiating between the two can be aided by detecting viral DNA in the joint fluid and identifying bacterial growth in joint fluid or blood cultures, along with high levels of LDH, protein, and acute phase reactants. Literature indicates that bacterial growth is detected in 30% of blood cultures and around 70% of joint fluid cultures in septic arthritis cases. The most frequent causative agent is S. aureus, followed by Group A streptococci, *Streptococcus pneumoniae*, and *Kingella kingae*.

Though primary viral agent-driven septic arthritis is rare, it can occur as an immune-mediated reaction, especially following live vaccines. In our case, the patient was empirically treated for septic arthritis based on Kocher’s Criteria, with symptoms including a high fever (38.5°C), resistance to walking, an increased WBC count (>12×10%/l), and an elevated ESR (>40 mm/hr). VZV PCR was not performed on the joint fluid, which appeared turbid yellow, and both blood and joint fluid cultures were negative. Empiric treatment included vancomycin (for 6 days) and ceftriaxone (for 4 days), as cefazolin or nafcillin are recommended when cultures are negative, with vancomycin or clindamycin used in regions with high methicillin-resistant agent prevalence.

### Purpura fulminans

Purpura fulminans is a rapidly progressing condition characterized by dermal hemorrhage resulting from intracapillary thrombus formation without histological vascular wall involvement. The condition begins suddenly with purplish-black areas of hemorrhagic cutaneous necrosis and deranged coagulation factors, carrying a high mortality rate. Visceral involvement is uncommon but can lead to hematuria and gastrointestinal bleeding.

Purpura fulminans occurs in three clinical settings:

1. Overwhelming sepsis: Various bacteria, including *Staphylococcus aureus*, groups A and B B-hemolytic streptococci, Streptococcus pneumoniae, Hemophilus influenzae, and meningococcal septicemia, have been associated with the disorder.
2. Neonates and infants with inherited deficiencies of protein C or protein S: Protein C cleaves activated cofactors V and VIII, reducing fibrin clot formation. Protein S, a cofactor of protein C, enhances its effects. Deficiencies in these proteins can lead to a hypercoagulable state responsible for thromboembolic events.
3. Idiopathic PF: Primarily occurs in children with rapidly progressive purpura after a febrile illness. The association with preceding infections suggests “postinfectious” rather than “idiopathic” as a more appropriate description. Varicella and streptococcal infections are the most common preceding infections, occurring in 30% and 20% of patients, respectively.

### Case comparison

In comparison to the literature, our case involves a 7-year-old male, which fits within the reported age range of 1.5–11 years. The affected joint, the left knee, is consistent with the most frequently reported joint (31%). Onset occurred two days after the varicella rash, within the reported range. The arthritis duration was less than one month, similar to 79% of reported cases. Laboratory values were within the reported ranges: WBCs at 14K (range 3.5K–16.7K), ESR at 20 (22% of reported values were less than 21), and synovial fluid WBCs at 14K (range 1.65K–73.5K) with lymphocytes at 20% (range 2–100%).

Regarding purpura fulminans, our case is unique in its spread pattern from the dorsum of the feet to the ankle and proximal tibia, whereas the most common locations in literature are the lower limbs (89–97%). PS activity levels were reduced, consistent with recent case series and literature. Treatment included heparin and FFP, similar to 100% and 97% of cases, respectively. Despite using iloprost, the outcome was amputation, the third most frequently reported outcome in the literature. Other outcomes included skin necrosis with grafting (28–29%) and venous thromboembolism (33–32%). Extensive lab tests indicated an acquired protein S deficiency caused by anti-protein-S antibodies. The improvement in protein S levels after receiving FFP, and normal protein S levels in the patient’s parents, support this diagnosis.

The presence of antiphospholipid antibodies in patients with purpura fulminans following varicella infection may explain the severity of clinical manifestations. This suggests that transient autoimmune-mediated protein S deficiency, potentially due to a cross-immune reaction between protein S and Varicella-Zoster virus, led to a hypercoagulable state. Prompt heparinization and large volumes of FFP were effective in halting disease progression, though protein S concentrate was ultimately needed to restore normal levels^26,27^.

## Conclusion

This case underscores the intricacies associated with varicella-related complications, exemplifying the unusual presentation of both septic arthritis and purpura fulminans in a pediatric patient. It underscores the significance of comprehensive comprehension and collaborative management strategies for prompt intervention and enhanced clinical results. Regrettably, in numerous countries where there is no vaccination against the chickenpox virus, we advocate for the administration of this vaccine to all infants to avert the devastating consequences of chickenpox, as witnessed in our patient.

## Ethical approval

The ethical approval was given by the ethical committee of AL Ahli Hospital and with the one in Hadassah Hospital via coordination with the head of Paediatric Surgery department.

## Consent

The consent was obtained and it included the declare of patient’s privacy protection.

## Source of funding

None.

## Author contribution

B.H., E.N. and R.M.Y. have all contributed equally in writing the paper. F.M.A. was the supervisor.

## Conflicts of interest disclosure

The authors declare no conflicts of interest.

## Research registration unique identifying number (UIN)

Case Report.

## Guarantor

All the authors.

## Data availability statement

Data sharing is publicly available.

## Provenance and peer review

Not published.
